# Does Learning Through Movement Improve Academic Performance in Primary Schoolchildren? A Systematic Review

**DOI:** 10.3389/fped.2022.841582

**Published:** 2022-03-08

**Authors:** Luca Petrigna, Ewan Thomas, Jessica Brusa, Federica Rizzo, Antonino Scardina, Claudia Galassi, Daniela Lo Verde, Giovanni Caramazza, Marianna Bellafiore

**Affiliations:** ^1^Sport and Exercise Sciences Research Unit, Department of Psychology, Educational Science and Human Movement, University of Palermo, Palermo, Italy; ^2^Regional School Office of Sicily (USR Sicilia), Palermo, Italy; ^3^Istituto Comprensivo Statale Giovanni Falcone, Palermo, Italy

**Keywords:** preschool, infant, kindergarten, outdoor learning, nature, academic achievement, primary school

## Abstract

Physically active children have greater motor competence and a faster maturation compared with their sedentary peers. Recent research also suggests that physical activity during childhood may also promote cognitive development and therefore improve academic performance. The aim of this study was to understand if physically active academic lessons may improve academic achievement in primary schoolchildren. A systematic review following the PRISMA (Preferred Reporting Items for Systematic Reviews and Meta-Analyses) guidelines was conducted. The search was performed on the following database: PubMed, Web of Science, Scopus, Education Resources Information Center (ERIC), and PsycINFO (APA). Studies evaluating schoolchildren aged between 3 and 11 years taking part in educational contexts that include physical activity and natural environments evaluating physical fitness and/or educational outcomes were included. A total of 54 studies (for a total sample of 29,460 schoolchildren) were considered eligible and included in the qualitative synthesis. The Effective Public Health Practice Project risk-of-bias assessment revealed a moderate quality of the included studies with only two considered weeks. Despite differences in the retrieved protocols, physically active academic lessons improve the total time engaged in physical activity, motor skills, and/or academic performance. The results of this review suggest that learning through movement is an effective, low-cost, and enjoyable strategy for elementary schoolchildren.

## Introduction

Children spend an ever-increasing time in sedentary behaviors such as the ~2 h (children aged 3 years) or 3 h (children aged between 3 and 5 years) per day in television view (1). Sedentary behaviors are also in school settings during which ~80% of the time children are seated (2), and only 5% of the time is spent in moderate to vigorous activities in European schoolchildren (3). Consequently, considering the classroom as a place where students spend the majority of their waking time, the school setting can be considered as an ideal setting to improve physical activity and academic achievement and also because it has positive results inside and outside the school (4, 5).

The key point to limit sedentary behaviors is to propose health promotion programs to promote physical activity since early childhood (6). Physical activity interventions for children should improve physical fitness, promote health-related behaviors, and facilitate mental development (7, 8). Physical activity, especially during development, has positive effects on the measures of adiposity, motor skill, bone and skeletal health, psychosocial health, cardiometabolic health indicators, and cognitive development (9–11). Gross and motor skill practice has also positive effects on cognitive development (12) and functions (such as perceptual skills, intelligence quotient, academic achievement and readiness, verbal and mathematics tests, developmental level) (13, 14), non-executive cognitive functions, core executive functions, and higher-level executive functions (15). It seems that aerobic training has the largest effects (16). Its performance during early childhood could become a lifelong habit, improving cognitive and physical health (17), making the physical movement even more important in this phase of life. Consequently, states need to monitor and evaluate strategies to increase physical activity during school time, adopting a policy specific to prevent potential loopholes (18). On the other side, elementary schools could be a platform for early intervention to improve daily physical activity, but further investigations are required to secure the successful assimilation of movement integration into routine practices (19). Especially in children, physical activity practice during school days can be incorporated, and it increases moderate to vigorous physical activity levels (20) and improve aerobic fitness (4) and also has positive learning outcomes and consequently academic achievement (21). It can integrate physical activity in the academic curriculum and consequently propose a classroom-based physical activity program, increase children's cognition (15) and energy expenditure (22), develop social skills, improve mental health, and reduce risk-taking behaviors, but it also has short-term cognitive benefits (23). A physical education program could be a decisive education strategy to enhance motor and cognitive learning in preschool children and to achieve successful academic outcomes (24). Physically active lessons can be proposed with different contents such as math, language, arts, and social sciences, and this has also positive effects on physical activity level and learning and attention (25). The inclusion of physical activity in the curriculum to improve learning outcomes is feasible, and it is suggested in elementary schoolchildren (21).

Schools and teachers are culturally changing, adopting active learning and other kinds of learning methods, but further improvement is required (26). Unfortunately, individuals and schools limit the application of these kinds of programs (27). In 2012, Erwin et al. (21) suggested that more research is required to study integrated physical activity interventions, both on the learning outcome and physical activity levels. Consequently, the objective of this systematic review was to analyze the protocols adopted and the effects of outdoor learning on schoolchildren.

## Materials and Methods

The systematic review was conducted following the principles outlined by PRISMA (Preferred Reporting Items for Systematic Reviews and Meta-Analyses) guidelines (28).

### Eligibility Criteria

The selection criteria of this review were of the PICO-S (Population, Intervention, Comparison, Outcomes, and Study) design.

The population was composed of young children aged between 3 and 11 years of primary/elementary schools. Studies that investigated only a special population such as people with disabilities were excluded because of the possible disability-specific outcomes.

The intervention of interest had to be the use of movement and natural environment with educational elements integrated to improve physical fitness and/or educational outcomes. Curriculum physical education, physical activity breaks without educational elements, recess, and after-school interventions were excluded.

The comparison and the outcomes of interest comprised physical fitness parameters and education outcomes.

About the study design, only English-written original and peer-reviewed studies were considered because of the limitations of the authors with the languages. Intervention, cross-sectional, longitudinal, correlational (randomized and non-randomized controlled, and quasi-randomized studies) studies were also included. Reviews, meta-analyses, abstracts and scientific conference abstracts, citations, opinion articles, books and book reviews, letters, editorials, statements, and commentaries were excluded.

### Data Collection

The systematic search was performed through the electronic databases PubMed, Web of Science, Scopus, Education Resources Information Center (ERIC), and PsycINFO (APA).

The following keyword groups were adopted and matched with the Boolean operators AND/OR:

Group 1: *child, preschool, infant, toddler, pupil, kindergarten*;
Group 2: *primary school, elementary school, student, education*;
Group 3: *psychomotor education, physical education, kinesiology education, active play, motor play, active learning, nature play, whole school, movement integration, comprehensive school, physical activity break*.

This is a string example:

(Child^*^ OR preschool^*^ OR infant^*^ OR toddler^*^ OR pupil^*^ OR kindergarten) AND (“primary school” OR “elementary school” OR student^*^ OR education) AND (psychomotor education OR physical education OR kinesiology education OR active play OR motor play OR nature play OR whole school OR movement integration OR comprehensive school OR physical activity break OR active learning).

### Study Record

The selected articles were included in EndNote software (EndNote version X8; Thompson Reuters, NY, USA). In the first step, duplicates were detected. After this step, two investigators, who worked independently, performed a selection process based on the inclusion and exclusion criteria on the title, abstract, and full-length articles. If the two investigators were in disagreement in categorizing an article, the coordinator of the study was involved and, independently, provided the tie-breaking decision. All investigators were not blinded to the authors or associated institutions of the articles during the selection process.

Information related to the sample (age, gender, and sample size) and intervention (type, duration, frequency) characteristics, and on physical fitness and educational outcomes was collected. The data were discussed narratively and represented through tables.

### Risk-of-Bias Assessment

To detect the risk of bias and the quality of the study, the Effective Public Health Practice Project tool (29) was adopted. This tool is composed of three scores (weak, moderate, or strong) that were assigned to the following: (1) selection bias assessment, (2) study design evaluation, (3) confounder factors, (4) blinding, (5) data collection methods, (6) withdrawals, and (7) dropouts, to provide an overall rating. A “strong” scoring was provided to a study if at least four strong ratings and no weak rating were provided to each sub-domain. A “moderate” scoring was provided to a study if it had less than four strong ratings and one weak rating provided to the subdomains. A “weak” scoring was provided to a study if two or more weak ratings were provided to the subdomains. In order to numerically quantify the subdomains, a score of 3 was attributed to a strong evaluation, a score of 2 was attributed to a moderate evaluation, and a score of 1 was attributed to a weak evaluation.

## Results

**Figure 1:**
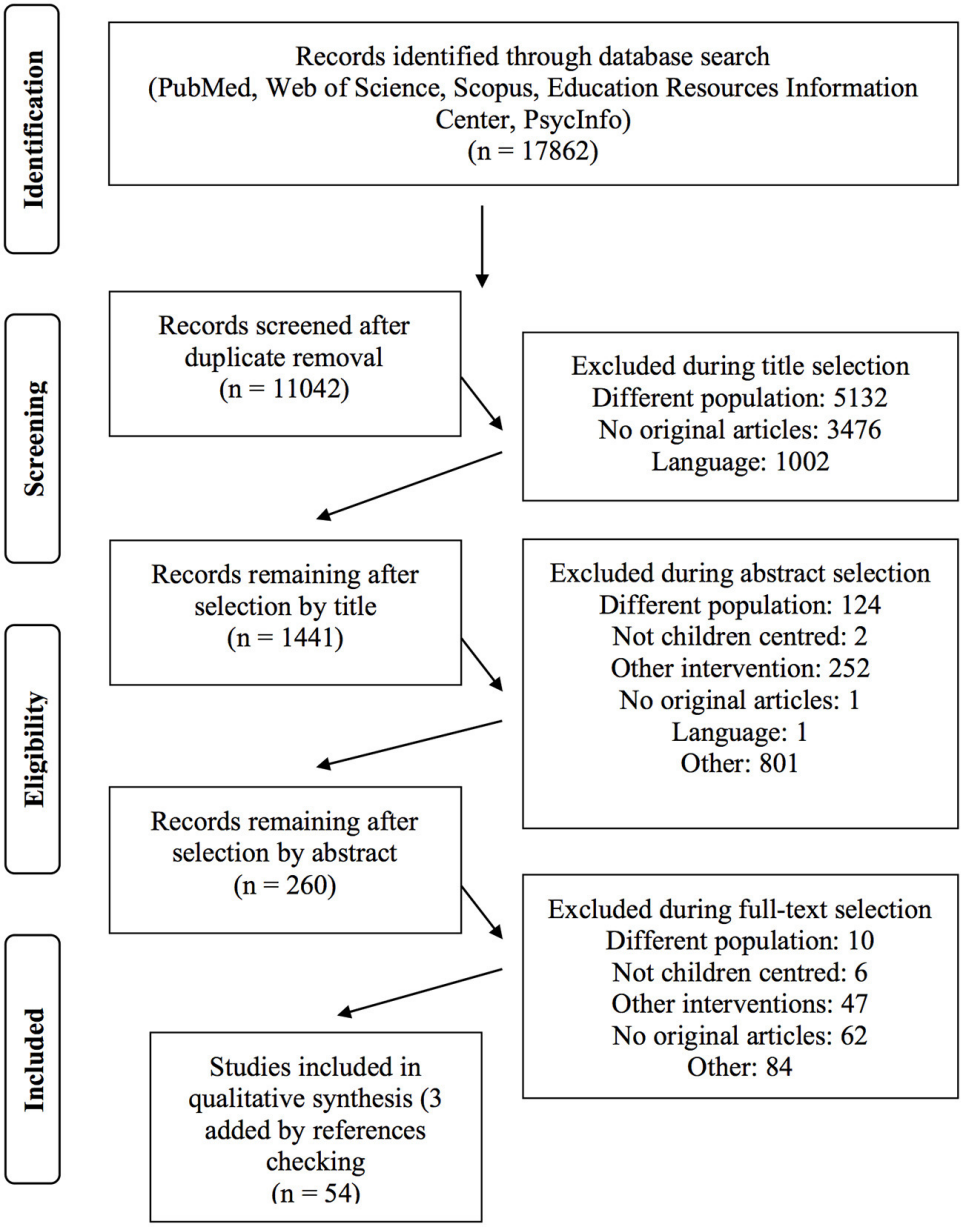
PRISMA flow chart illustrating the systematic process.

A total of 17,862 studies were found in the electronic databases searched, and 6,820 of the articles were immediately removed because they were duplicates. The final number of included studies after the eligibility criteria screening has been of 54 (three studies were included in a second moment after the reference checking of the included studies). A summary of the search process is provided in Figure 1.

### Risk of Bias

The quality of the studies was overall moderate, with only two studies deemed weak. The mean score for selection bias was 3/3, for the study design 2.5/3, for the confounder 2.4/3, for the blinding of 1.3/3, for the data collection of 2.2/3, and for the dropout of 2.9/3, reaching an overall mean total score of 1.8 out of 3.

### Study Characteristics

A summary of the study's characteristics is proposed in Table 1. The number of participants included in the studies was 29,460; one study did not specify the number of students, but the number of classrooms included was 4. A total of 11,392 were composed of girls (39%), 11,021 were boys (38%), whereas in 6,486, the gender was not specified (23%). The mean age (standard deviation) of the included participants was 8.2 (0.7) years, and it ranged from 3.9 to 11.2 years.

**Table 1:** Main descriptive characteristics of the included studies.

**References**	**Nationality**	**Study design**	**Sample size (female) [male]**	**Mean age (standard deviation)**	**Intervention**	**Length (days)**	**Duration (min)/times a week**
Aadland et al. (30)	Norway	RCT	1,129 (541) [588]	10.2 (0.3)	Active learning	12	30/3
Alhassan et al. (31)	USA	RCT	67 (29) [38]	4 (0.7)	Active school	28	30/3
Bacon and Lord (32)	United Kingdom	RCT	36 (15) [21]	9.5	Active learning	14	10/5
Bai et al. (33)	Australia	Observational	1,596	3.5	Play and learning	1,095	No info
Bartholomew et al. (34)	USA	RCT	2,716 (1,467) [1,249]	9.5	Active learning	5	15/5
Bartholomew et al. (35)	USA	RCT	2,493 (1,349) [1,144]	9	Active learning	No info	15
Braun et al. (36)	USA	Observational	3,479	9.5	Active learning	365	No info
Brusseau et al. (37)	USA	Quasi-experimental	1,460 (730) [730]	8.4 (1.8)	Active classroom	84	50
Bugge et al. (38)	Denmark	Quasi-experimental	1,181 (629) [552]	8.4 (1.4)	Active classroom	No info	60/6
Burns et al. (39)	USA	Quasi-experimental	327 (162) [165]	9.6 (1.7)	Integrated PA	84	No info
Burns et al. (40)	USA	RCT	1,460 (730) [730]	8.4 (1.8)	Integrated PA	No info	No info
Christodoulos et al. (41)	Greece	RCT	68 (36) [42]	11.2 (0.3)	Active classrooms	No info	45/2
Cradock et al. (42)	USA	Quasi-experimental	393 (206) [187]	10.2 (0.8)	Active classrooms	150	No info
Dyrstad et al. (43)	Norway	RCT	483	9.5	Active school	238	45/2
Egan et al. (44)	USA	Quasi-experimental	161 (78) [83]	7.3 (0.9)	Active learning	120	No info
Goh et al. (45)	USA	Observational	210 (118) [92]	9.1 (0.1)	Active classroom	28	10
Grieco et al. (46)	USA	Mixed factorial	320 (164) [156]	8	Physically active lessons	No info	15
Invernizzi et al. (47)	Italy	RCT	121 (64) [57]	10.5 (0.5)	Active classroom	84	60/2
Konijnenberg and Fredriksen (48)	Norway	Quasi-experimental	1,173 (595) [578]	10.2 (1.5)	Active classroom	210	45/6
Martin and Murtagh (20)	Ireland	Pilot study	28 (14) [14]	8.5	Integrated PA	No info	No info
Martin and Murtagh (49)	Ireland	RCT	248	10	Integrated PA	5	No info
Mattson et al. (50)	USA	Observation	789 (355) [434]	10	Active classroom	No info	No info
Mavilidi et al. (51)	Australia	Mixed experimental	125 (61) [64]	4.9 (0.6)	Integrated PA	28	15/2
Mavilidi et al. (52)	Australia	Intervention	90 (45) [45]	4.9 (0.6)	Integrated PA	28	15/2
Mavilidi et al. (53)	Australia	RCT	120 (57) [63]	4.7 (0.5)	Integrated PA	28	15/2
Miller et al. (54)	Australia	RCT	168	11.2 (1)	Game intervention	49	No info
Mullender-Wijnsma et al. (55)	Netherlands	Within subject	81 (41) [40]	8.2 (0.6)	Active learning	154	30
Mullender-Wijnsma et al. (56)	Netherlands	Quasi-experimental	228 (106) [122]	8.1	Active learning	154	30
Norris et al. (57)	United Kingdom	RCT	264	8.5	Active classroom	42	10/3
Norris et al. (58)	United Kingdom	RCT	219 (108) [111]	8.6 (0.5)	Active classroom	42	10/3
Oliver et al. (59)	New Zealand	Intervention	78 (41) [37]	9	Integrated PA	28	No info
Pham et al. (60)	Vietnam	Pedagogical experiments	55 (32) [23]	7	Active school	150	35/2
Powell et al. (61)	United Kingdom	Quasi-experimental	485	8	Active learning	No info	No info
Powell et al. (62)	United Kingdom	Quasi- experimental	84	8.5	Active learning	No info	No info
Reed et al. (63)	USA	RCT	155 (67) [88]	9.5	Active learning	120	30/3
Resaland et al. (64)	Norway	RCT	1,129 (542) [587]	10.2 (0.3)	Active learning	310	No info
Resaland et al. (65)	Norway	RCT	1,129 (542) [587]	10.2 (0.3)	Active learning	310	No info
Riley et al. (66)	Australia	Pilot study	54 (26) [28]	10.5 (0.7)	Active learning	42	60/3
Robinson et al. (67)	USA	RCT	72 (37) [35]	3.9 (0.2)	Active classroom	63	30/2
Ruiter et al. (68)	Netherlands	RCT	118 (71) [47]	7.1 (0.4)		No info	No info
Schneller et al. (69)	Denmark	Quasi-experimental	361 (140) [221]	10.9 (1)	Movement integration	No info	45
Schneller et al. (70)	Denmark	Quasi-experimental	663 (317) [346]	10.8 (1)		No info	45
Seljebotn et al. (71)	Norway	RCT	447 (219) [228]	9.5	Games intervention	300	No info
Trawick-Smith et al. (72)	USA	Intervention	47 (27) [20]	3.9 (0.6)	Play to learn	No info	No info
Vazou et al. (73)	USA	RCT	77 (36) [41]	9.4 (0.5)	Active learning	56	10
Vazou et al. (74)	USA	Quasi-experimental	245 (105) [140]	5.7 (1.4)	Active learning	49	No info
Vetter et al. (75)	Australia	RCT	172 (89) [83]	8.4 (0.3)	Active learning	42	30/3
Vetter et al. (76)	Australia	RCT	85 (38) [47]	9.8 (0.3)		42	30/3
Weaver et al. (77)	USA	Quasi-experimental	1,826 (1,029) [797]	7.5	Integrated PA	730	No info
Weaver et al. (78)	USA	Intervention	229 (104) [125]	7.3 (0.8)	Active classroom	No info	10
Webster et al. (79)	USA	Mixed methods	4 Classrooms	7	Active learning	730	No info
Williams et al. (80)	USA	Pilot observational	207	4.6	Movement integration	10	10
Zachopoulou et al. (81)	Greece	RCT	251 (121) [130]	4.3 (0.5)	Active learning	70	40
Zippert et al. (82)	USA	Observational	251 (121) [130]	4.3 (0.5)	Play intervention	No info	20

The studies were performed in different countries. The majority of the studies were performed in the United States (*n* = 22). In Australia, a total of nine studies were conducted. Five studies were conducted in the United Kingdom and Norway. More than one study was conducted in Denmark (*n* = 4), in the Netherlands (*n* = 3), in Greece (*n* = 2), and in Ireland (*n* = 2). Only one study was conducted in Italy, New Zealand, and Vietnam.

The majority of the studies were randomized controlled trials (*n* = 25). They were followed by quasi-experimental design (*n* = 13), observational studies (*n* = 5), intervention studies (*n* = 4), and pilot studies (*n* = 3). Other study designs such as mixed factorial, mixed experimental, within subject, and pedagogical experiments were adopted only one time.

Seven interventions provided negative feedback on the effect of integrated lessons on physical activity and/or academic outcomes, and there are no aspects between the studies that could suggest excluding some aspects of the intervention such as the duration of the program or session, or the kind of intervention, or the subject considered.

### Intervention Characteristics

Different studies were based on national or international intervention programs. The Comprehensive School Physical Activity Programs was the intervention program adopted majority of times (*n* = 4). Adopted in three different studies is the Active Smarter Kids intervention. Less adopted assessment methods are provided in Table 2.

**Table 2:** Synthetic description of the interventions included.

**References**	**Intervention**	**Subjects**	**Academic evaluation**	**Physical assessment**	**Conclusion**	**Effect on PA**
Aadland et al. (30)	ASK	Norwegian, math, English	Executive functions	Acc; executive functions; Andersen test; motor skills	Small effects on executive functions, cognitive flexibility	0% (MVPA)
Alhassan et al. (31)	SPARK	No info	No info	Acc	Improvements in PA	+22.8% (MVPA)
Bacon and Lord (32)	No info	Math	No info	Acc	Improve PA and academic outcomes	+22-5% (steps)
Bai et al. (33)	PLAYCE	No info	No info	No info	Improve educators' self-efficacy to engage in PA	ND
Bartholomew et al. (34)	I-CAN!	Math, language arts	Time on task	Acc	Significantly increased time on task	+43.6% (MVPA)
Bartholomew et al. (35)	I-CAN!	Math, language arts	No info	Fitnessgram	Increases PA within elementary students	ND
Braun et al. (36)	CSPAP	Math	No info	PACER	Need for more prospective research	+19% (min/week)
Brusseau et al. (37)	CSPAP	No info	No info	Acc, Fitnessgram; PACER	Improve PA	+17.9% (MVPA)
Bugge et al. (38)	CHAMPS	Math, Danish	Academic achievement	Andersen test	No negative effects of additional PA on scholastic outcomes	ND
Burns et al. (39)	CSPAP	No info	No info	Acc	Increase PA	+26.2% (steps)
Burns et al. (40)	CSPAP	No info	No info	TGMD-2	Motor skills improved	ND
Christodoulos et al. (41)	No info	Math, reading, handicraft	No info	20-m shuttle run; sit and reach, sit-up test	Slow the age-related decline in PA	ND
Cradock et al. (42)	SPARK	No info	No info	Acc	Increase moderate to vigorous PA	+45.7% (MVPA)
Dyrstad et al. (43)	No info	Language, math	No info	No info	Appropriate pedagogical method	ND
Egan et al. (44)	PACES	Math	No info	SOFIT	Effectiveness of the research	ND
Goh et al. (45)	TAKE 10!^®^	Language arts, math, science, social studies, general health	No info	Pedom	Improvement of children's PA	+15% (steps)
Grieco et al. (46)	No info	No info	Time on task	Acc	PA increases time on task	+96.9%(MVPA)
Invernizzi et al. (47)	No info	No info	No info	PAQ-C; MFT; TGMD-2; PACES	Positive effects on physical literacy development	ND
Konijnenberg and Fredriksen (48)	HOOP	Language, math	Stroop/Eriksen, flanker tasks	No info	No positive effect of the PA intervention	ND
Martin and Murtagh (20)	No info	English, math	No info	Acc	Improve PA	+96.2% (MVPA)
Martin and Murtagh (49)	No info	No info	No info	Acc	Improve PA	+4.2% (MVPA)
Mattson et al. (50)	CSPAP	English, math	No info	No info	Increase PA	ND
Mavilidi et al. (51)	No info	No info	Recall words, free recall, cued recall	Acc	Highest learning outcomes	+54.5% (MVPA)
Mavilidi et al. (52)	No info	Geography	No info	Acc	Positive way to increase learning	+41.9% (MVPA)
Mavilidi et al. (53)	No info	Math	Cognitive task	Acc	Improve math learning	+55.4% (MVPA)
Miller et al. (54)	PLUNGE	No info	Time on task	Pedom; TGMD-2	Improve object control motor skills proficiency and PA	+95.9% (steps/min)
Mullender-Wijnsma et al. (55)	F&V	Math, language	Time on task	20-m shuttle run test	Positively influence time on task	ND
Mullender-Wijnsma et al. (56)	F&V	Math, language	Time on task, Tempo-Test- Rekenen, Eén-Minuut-Test	No info	The lessons contributed to the academic outcomes	ND
Norris et al. (57)	Virtual Traveler	Math, English	No info	No info	Low- cost PA intervention	+7.7% (MVPA)
Norris et al. (58)	Virtual Traveler	No info	No info	Acc	Integrated PA has no negative effects on education	ND
Oliver et al. (59)	No	English, social studies, math, statistics	No info	Pedom	Significant increases in step counts	ND
Pham et al. (60)	BRAINball	Language, math, history, geography, biology	No info	TGMD-2	Positive effect on children's motor performances	ND
Powell et al. (61)	SHARP	No info	No info	SOFIT	Significant increases in PA	+4.1% (MVPA)
Powell et al. (62)	SHARP	No info	No info	SOFIT	Effective teaching strategy	+37% (MVPA)
Reed et al. (63)	No info	Language arts, math, and social studies	Fluid intelligence Academic performance	Pedom	Movement can influence fluid intelligence	ND
Resaland et al. (64)	ASK	Norwegian, math, English	Academic performance	Acc	No evidence to affirm the correlation	+3.4% (MVPA)
Resaland et al. (65)	ASK	Norwegian, math, English	Academic performance	Acc	Increase in academic performance	ND
Riley et al. (66)	EASY Minds	Math	On-task behavior	Acc	Improve on-task behavior in mathematics lessons	+3% (MVPA)
Robinson et al. (67)	CHAMP	No info	No info	SOFIT	Increase in PA	+9.1% (MVPA)
Ruiter et al. (68)	No info	Math	Math test, Evaluation Questions	No info	Movement conditions increase test results	ND
Schneller et al. (69)	EOtC	Math, history, language, religion	No info	Acc	Time- and cost-neutral increase time spent in PA for boys	+7.5% (MVPA)
Schneller et al. (70)	EOtC	No info	No info	Acc	Opportunity to accumulate PA	+8.4% (MVPA)
Seljebotn et al. (71)	Active school	Several subjects	No info	Acc	Increased PA	+13% (MVPA)
Trawick-Smith et al. (72)	No info	Math	TEMA-3, Communication about math	Food-fit play interactions	Teacher interactions in children's play help academic results	ND
Vazou et al. (73)	Move 4 Thought	Math	No info	Acc	Contribute to increasing PA levels	+60.6% (MVPA)
Vazou et al. (74)	Walkabouts	Math, language arts	No info	SOSMART	Academic does not impact PA	ND
Vetter et al. (75)	Maths on the move	Math	NAPLAN	Acc; shuttle run test	Improve of learning and PA	+92.7% (MVPA)
Vetter et al. (76)	No info	No info	Numeracy	Aerobic fitness	Positive combination of PA with learning	ND
Weaver et al. (78)	PACES	No info	No info	Acc	Routine practice increase PA	+5.6% (MVPA)
Weaver et al. (79)	PACES	No info	No info	Acc	Increase PA	+1.8% (MVPA)
Webster et al. (79)	PACES	No info	No info	No info	No impact	ND
Williams et al. (80)	Animal Trackers	No info	No info	No info	Increased structured PA	ND
Zachopoulou et al. (81)	Active learning	Math	TCAM test	No info	Improve creative fluency, imagination	ND
Zippert et al. (82)	Play	Math	TEMA-3, PPVT-IV	No info	Play improve math	ND

*Acc, Accelerometer; ASK, Active Smarter Kids; CHAMPS, Childhood Health, Activity, and Motor Performance School Study; CSPAP, Comprehensive School Physical Activity Program; EASY, Encouraging Activity to Stimulate Young; EOtC, education outside the classroom; F&V, Fit and Academically Proficient at School; HOPP, Health Oriented Pedagogical Project; MVPA, moderate to vigorous physical activity; MFT, Multistage Fitness Test; PACER, Progressive Aerobic Cardiovascular Endurance Run; PACES, Partnerships for Active Children in Elementary Schools; PA, physical activity; PAQ-C, Physical Activity Questionnaire for Older Children; Pedom, pedometer; PLAYCE, Play Spaces and Environments for Children's Physical Activity; ND, no data; SPARK, Sports, Play, and Active Recreation for Kids; I-CAN!, Texas Initiatives for Children's Activity and Nutrition; SOFIT, System for Observing Student Movement in Academic Routines and Transitions; SOSMART, System for Observing Student Movement in Academic Routines and Transitions; TGMD-2, Test of Gross Motor Development 2*.

Most of the interventions wanted to improve mathematics (*n* = 30) and language (*n* = 14) learning. Language arts was proposed as integrated lessons in five studies, social studies in only three studies, and two times for geography and history. Other subjects such as reading, handicrafts, science, general health, statistics, biology, and religion were studied only one time. In different studies, no information related to the curriculum subjects studied has been provided (*n* = 22).

The mean length of the intervention was 153.5 days, with a range from 5 to 1,095 days. The mean duration of the integrated physical activity was of 28.5 min, with interventions that were of 10 and others arrived to 60 min. Different studies proposed three interventions a week (*n* = 10), but other studies proposed only 2 days a week of curriculum-integrated physical activity (*n* = 7). Five studies proposed more than 3 days a week of intervention (*n* = 5). Unfortunately, the majority of the studies (*n* = 32) have not provided this information.

The majority of the 29 studies (Table 2) that included data related to the physical activity level collected with accelerometers or pedometers had positive results, with a percentage range of improvement from 1.8 to 96.2. Only one study reported no improvement with the integrated movement program. Unfortunately, the data are not heterogeneous; indeed, studies compared different groups or the same group before and after the intervention. Studies reported the time in which the children were engaged in moderate to the vigorous physical activity or the number of steps. Studies collected data during the school hours or during the week or the day.

Academic achievements or cognitive functions were assessed majority of times through the academic outcomes and the time on task (*n* = 3). In three studies, the authors evaluated them through the “on-task” behavior. Less adopted assessment methods are provided in Table 2.

Related to physical activity assessment, 29 studies evaluated it through an accelerometer or a pedometer. Some studies evaluated health-related physical fitness characteristics through physical tests such as the Test of Gross Motor Development 2 (*n* = 4), Andersen test (*n* = 2), 20-m shuttle run test (*n* = 3), and Progressive Aerobic Cardiovascular Endurance Run (*n* = 2). Less adopted evaluation methods are provided in Table 2. Skill-related physical fitness was evaluated through test to evaluate executive functions and motor skills (*n* = 1). Studies adopted also batteries to evaluate physical fitness such as the FITNESSGRAM (*n* = 2). The most interesting subjective physical activity evaluation methods were the System for Observing Student Movement in Academic Routines and Transitions (*n* = 3), Physical Activity Questionnaire for Older Children (*n* = 1), interviews, and observations.

### Intervention Proposal

Some studies reported the intervention in detail or examples of intervention, and the following are proposals of the included studies. Some studies proposed outdoor structured nature-based play (33, 71) or adopted the outdoor environment to learn math, language, history, or religion (70). Games-centered interventions (34, 35) or games related the pedometer with mathematics (36) or free play or semistructured physical activity have been proposed (39). Always through play was the intervention of Pham and colleagues, which adopted balls with numbers, letters, and mathematical symbols on the surface (60). Complex, independent, and symbolic play (72) and playing with math-related materials to examine children's verbal and non-verbal mathematics exploration without adult guidance (82) were also proposed. Other proposals that comprised cooperative activities integrate health education into several school subjects (41). An intervention proposed as language activity “Scrabble relay,” where children worked in groups, or “Bingo” to improve mathematics (43). In another study, one teacher read a story while students perform the movements in the story (45). Mavilidi and colleagues proposed different interventions for different subjects. To learn language, children enacted the actions indicated by the words to be learned by physically exercising (i.e., for the word “fly,” children ran and moved their hands as if they were flying) (51). To learn geography, children “traveled” from one continent to the other, imitating the movements of the animal representing the continent (52). To learn math, foam blocks of numbers were placed on the floor, shaping a straight line, and the children ran, jumped, and stepped each time on one number while counting or walked or ran backward, sideward, or forward (53). A similar intervention to learn geography was the one proposed by Oliver et al. (59). Norris et al. (57) proposed in their intervention presentation sessions known as Virtual Field Trips, designed to be delivered using existing classroom interactive whiteboards. Similarly, children autonomously navigated through two skill stations with at least three levels of difficulty at each station (67). Other language and mathematics interventions consisted in the performance of a spell by jumping in place for every mentioned letter or to jump to solve multiplications. Similar academic tasks with different words or sums were exercised during one lesson (55, 56). Other interventions consisted in building two-digit numbers by making and simultaneously verbalizing out loud different-sized steps (68). Students stand on their self-space and jump the answer to a problem the teacher provided and the second by moving around the classroom, picking a card with a problem working as a group or with a partner (73). Locomotor skills of running, skipping, hopping, and galloping (75) integrating structured movement and motor skill practice with preschool learning concepts and integrating auditory, visual, and kinesthetic learning methods (80) were also proposed. Use and modification of movement elements, development of creative thinking during movement activities through exploration, use of movement for experienced learning of concepts of different teaching thematic areas such as mathematics, and development of critical thinking during movement activities were also adopted (81).

## Discussion

The findings of the review highlighted that different interventions were proposed to teach different curriculum subjects through movement with a lack of standardization in the protocols adopted by the authors (Table 2).

Similar to the findings of Erwin and colleagues, physical activity integrated in the academic curriculum is proposed with other interventions (such as breaks), and the details on the effect on children's learning and physical fitness are not always provided (21). It is important to propose a structured intervention; only in this way that it is possible to contextualize and generalize the finding and make the procedure safer (83), and the teachers have a crucial role in following the procedures proposed (22). Differences were also detected in the length, duration, and week frequency, making impossible a comparison among the studies. These findings are similar to the study by Daly-Smith and colleagues, where differences in the design, interventions, duration and intensity, and outcomes were detected (84). The intervention duration in this study started from 5 days arriving to more 1,000 days, differently from other studies in which the intervention ranged from 13 to 300 days (21). Even if the literature suggests that the length of the intervention did not influence the effect of the intervention (21), a short-duration program is not useful to have a long-term improvement on academic performance (85). Furthermore, it is important to propose the integrated programs in daily or weekly schedule because it increases also the physical activity during the school day, and it is feasible (86). Ideally, the physical activity interventions should be three times per week to obtain the best results on children's cognitive and achievement outcomes (16).

Differences were detected also in the interventions. The movement integration program wants to teach students through the movement. It is well-known that physical activity interventions have a positive effect on cognitive performance and academic performance in children (87, 88). Integrated physical activity in the classroom can increase children's academic intrinsic motivation, perceived competence, and effort without influencing academic lessons (89).

For those studies that proposed play as an intervention, positive outcomes have been detected. Learning through play forces children to make choices and assuming responsibility having fun at the same time, working on the internal cognitive transactions and intrinsic motivation, determine life habits (90). Play should have to be enjoyable, freely chosen, non-literal safe, and actively engaged; only in this way that learning is through intrinsic motivation (90).

Other interventions, instead, were based outdoors. This way of learning can be incorporated within conventional teaching methods (91); it increases physical activity and reduces sedentary behaviors (92). Open learning environments want to educate the students with own initiative, planning, experimentation, elaboration, and self-evaluation, which is an interesting way (26).

The interventions showed improvements in the academic outcomes, motor skills, or amount of physical activity (through step count), but an important point is that they are cost-effective, teachers are not required to prepare them, and they are enjoyable both for teachers and children (5), making them ideal for primary schools. The advantage of a classroom-based physical activity program integrated in the school curriculum is that it takes time from other subjects, but improves physical activity and on-task behavior without sacrificing or influencing academic performance (93, 94). Furthermore, physical education in elementary school children has no negative effects on standardized academic achievement test scores (95). Indeed, physical activity improves mathematics-related skills, reading, and composite scores such as the classroom behaviors, suggesting physical exercise lessons in the curriculum and physical activity integration in classroom lessons (96). Even brief bouts (1 h long) of outdoor active play can improve on-task behavior (97). The level of physical activity enjoyed outdoors on the playground is higher, and the increase in on-task classroom behavior is greater; simple play outdoors seems to be not sufficient (97). Physical activity incorporated into the school day improves attention to task (98). Physical, active academic lessons have several benefits for schools and students; indeed, they are cost-effective. Children and teachers enjoy them. They do not require additional teacher preparation time and improve academic achievement scores (5).

### Limitations and Future Studies

Data obtained from accelerometers were not analyzed because of the limitation of this tool in detecting activities performed with the upper body (30). Furthermore, the studies included in the review present a wide variety of testing conditions and interventions, making the performance of a meta-analysis impossible. The sample background (physical activity participation outside the school, social status, or other influencing factors) was not detected, making the comparison even harder.

The study has been focused only on a specific population. It has been suggested by the literature (16) that children with learning disabilities also present improvements in academic abilities when physical activity interventions are adopted, making the study of these interventions also in this population even more important. There is a lack of heterogeneity among the study interventions, with differences not only in the length of the program, duration of the session, and frequency but also in the intervention methodology and in the subjects included in the programs. Differences were also in the outcome studies, both for physical activity and academic performance evaluation. Future studies should focus their attention on review of the literature about physical activity breaks during classroom time to improve physical fitness and academic performance. Attention should be focused also on interventions performed in nature, for two reasons: first, the intervention moves the children outside, and second, this intervention can help the children to understand the importance of nature.

## Conclusion

All the interventions, despite differences in the protocols, have a common aspect: they improve physical activity and/or academic performance, making this kind of approach ideal in elementary schools.

## Data Availability Statement

The original contributions presented in the study are included in the article/supplementary material, further inquiries can be directed to the corresponding author.

## Author Contributions

GC and MB: conceptualization. CG: methodology. JB and FR: investigation. DLV: resources. LP: writing—original draft. ET and AS: writing—review and editing. MB: supervision. All authors contributed to the article and approved the submitted version.

## Funding

This publication has been funded by Assessorato dell'Istruzione e della Formazione Professionale dlla Regione Sicilia within the Natural Moving project.

## Conflict of Interest

The authors declare that the research was conducted in the absence of any commercial or financial relationships that could be construed as a potential conflict of interest.

## Publisher's Note

All claims expressed in this article are solely those of the authors and do not necessarily represent those of their affiliated organizations, or those of the publisher, the editors and the reviewers. Any product that may be evaluated in this article, or claim that may be made by its manufacturer, is not guaranteed or endorsed by the publisher.
